# Supplementary material for: Transcriptomic investigation of the interaction between a biocontrol yeast, Papiliotrema terrestris strain PT22AV, and the postharvest fungal pathogen Penicillium expansum on apple
Source: Commun Biol. 2024 Mar 22;7:359. doi: 10.1038/s42003-024-06031-w (PMC10960036; doi:10.1038/s42003-024-06031-w)
Supplement: Supplementary file 2 — Supplementary Information [file 42003_2024_6031_MOESM2_ESM.pdf]

Transcriptomic investigation of the interaction between a biocontrol yeast, *Papiliotrema terrestris* strain PT22AV, and the postharvest fungal pathogen *Penicillium expansum* on apple

Giuseppe Ianiri[1], Giuseppe Barone[1], Davide Palmieri[1], Michela Quiquero[1], Ilenia Gaeta[1], Filippo De Curtis[1], Raffaello Castoria[1]

[1] *Department of Agricultural, Environmental and Food Sciences, University of Molise, via F. De Sanctis snc, 86100 Campobasso, Italy.*

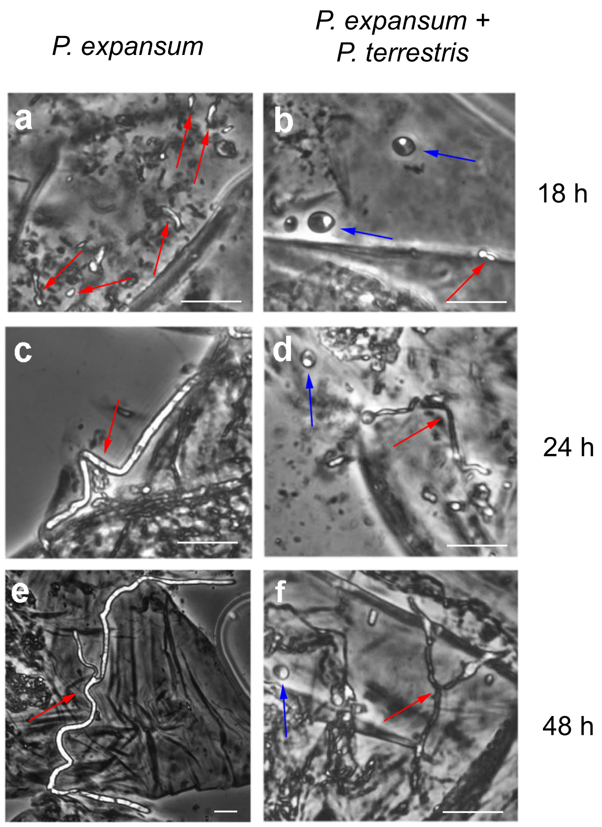

Supplementary Figure 1. Microscopic analysis to determine the time point for RNA extraction of the BCA *P. terrestris* and the fungus *P. expansum* during their dual and tritrophic interactions with the host *M. domestica*.

Pictures of *M. domestica* tissues at 18 hpi (a, b), 24 hpi (c, d) and 36 hpi (e, f) inoculated with *P. expansum* (a, c, e) and with *P. terrestris* and *P. expansum* (b, d, f). Red arrows indicate *P. expansum*, while blue arrows indicate *P. terrestris* yeast cells. Scale bar = 25 µm.

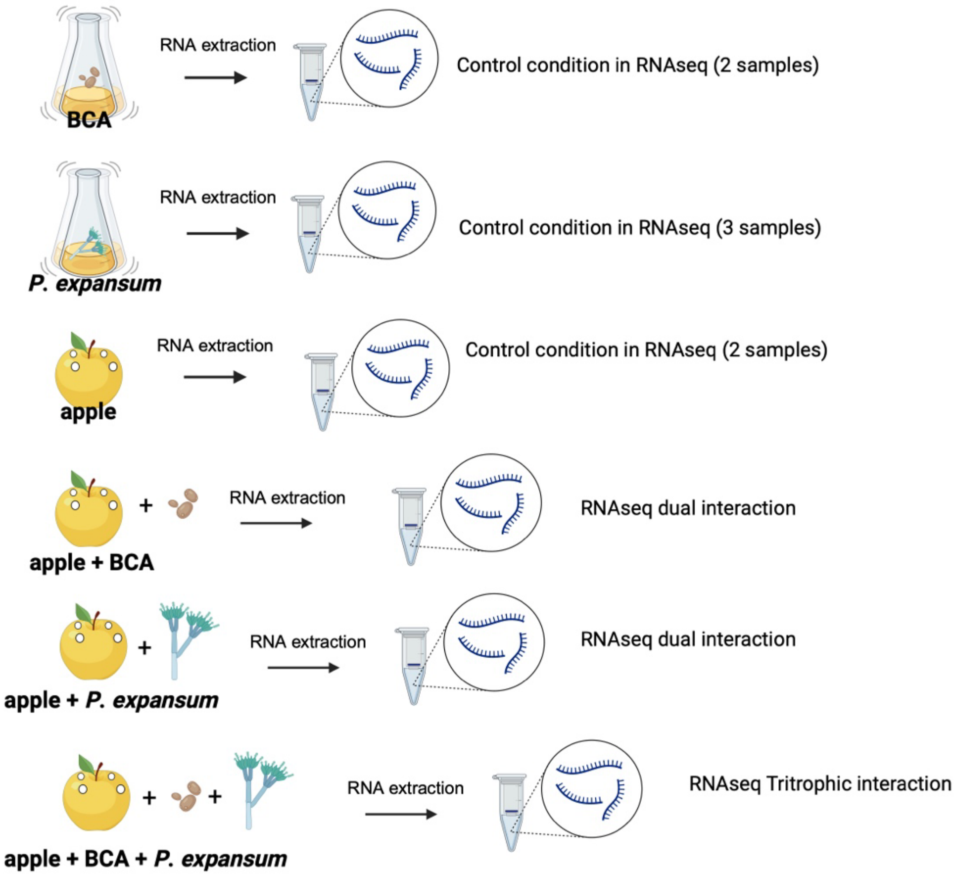

Supplementary Figure 2. Schematic representation of the experimental design used to perform the RNAseq analysis.

For all the conditions three biological replicates were predisposed, with the exception of uninoculated apple and *P. terrestris* grown *in vitro* that consisted of two biological replicates. This figure was created with BioRender.com. The figure was exported on a paid subscription.

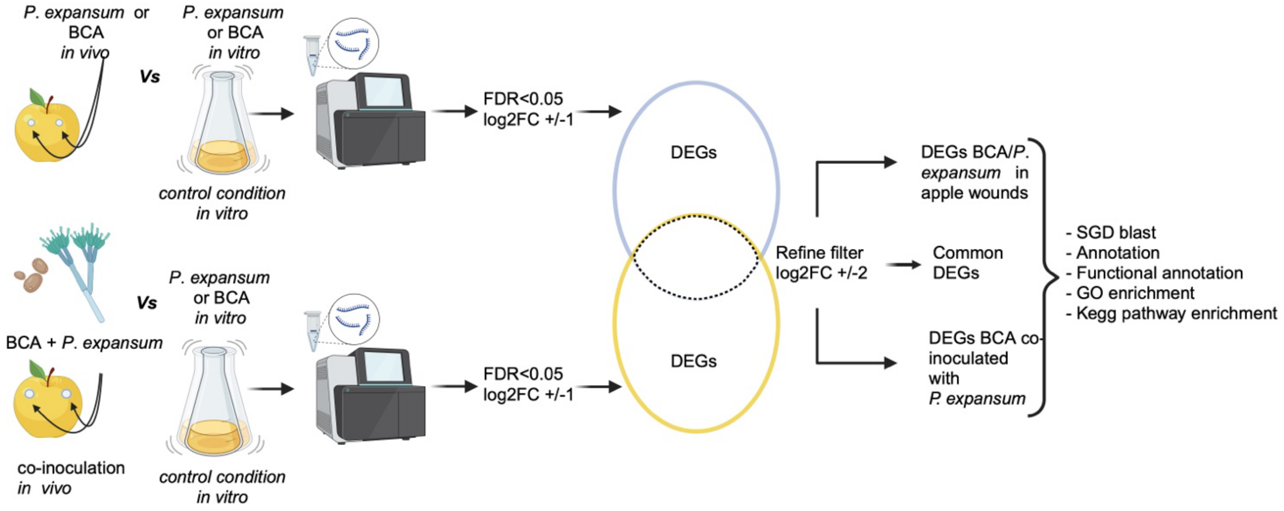

Supplementary Figure 3. Schematic representation of the pipeline used to obtain the final list of DEGs for *P. terrestris* and *P. expansum*.

As a first step, all genes with a FDR <0.05 and with a log2FC +/- 1 were selected; subsequently, results within each reference organism were compared with each other using the package VennDiagram, resulting in an additional datasets that include genes in common between the two conditions of study. As a last step, a further filtering step was carried out to select as DEGs those having a log2FC +/- 2. The function of the *P. terrestris* and *P. expansum* DEGs was inferred by BLASTp against *S. cerevisiae* (SGD, https://www.yeastgenome.org), and by gene ontology (GO) and by KEGG pathway enrichment. This figure was created with BioRender.com. The figure was exported on a paid subscription.

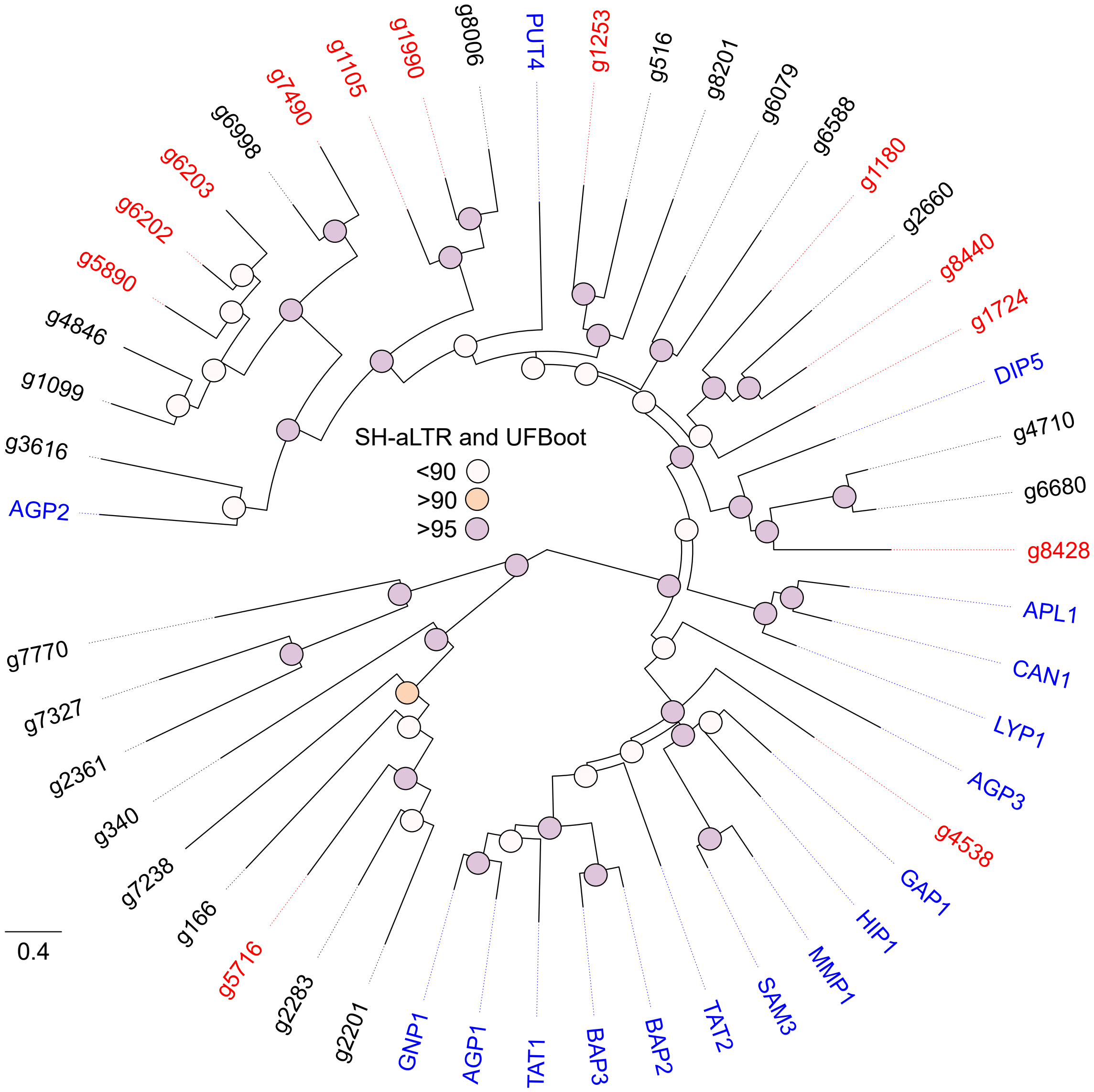

Supplementary Figure 4. Phylogenetic analysis of *P. terrestris* predicted amino acids transporters.

Maximum likelihood (ML) phylogenetic tree of the predicted aminoacid permeases of *P. terrestris* and *S. cerevisiae*. *Saccharomyces cerevisiae* permease sequences were identified according to the review of Bianchi et al., (2019) [1] and retrieved from the *Saccharomyces* genome database, while those of *P. terrestris* were identified either through BLAST analyses or from the genome annotation, and retrieved from the *P. terrestris* genome. The ML tree was generated using IQtree version 1.6.12 and the LG+F+R4 model, with support values were obtained from 1000 replicates of the Shimodaira–Hasegawa approximate likelihood ratio test (SH-aLRT) and ultrafast bootstrap (UFboot). Branches support is indicated. In blue are indicated *S. cerevisiae* transporters, in red transporters of *P. terrestris* upregulated during dual and tritrophic interaction with the host and the fungus *P. expansum*, and in black other *P. terrestris* transporters that are not upregulated in the RNAseq analysis.

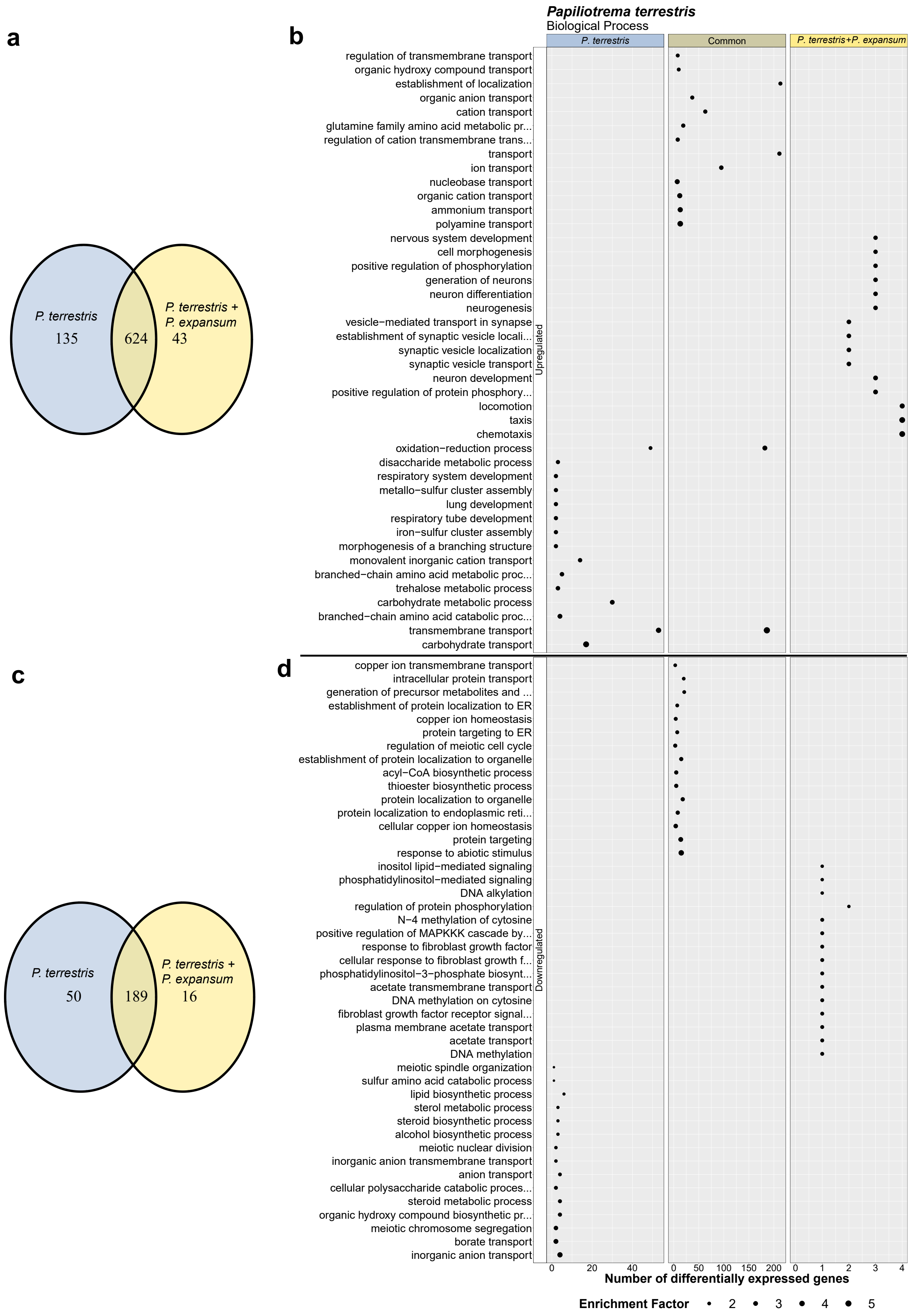

Supplementary Figure 5. Gene ontology analysis relative to the RNAseq analysis of the BCA *P. terrestris*.

(a - c) Venn diagrams showing common and unique sets of upregulated (a) and downregulated (c) genes of the BCA *P. terrestris* during dual and tritrophic interaction with the host *M. domestica* in the absence and in the presence of fungus *P. expansum,* respectively; the comparative control condition is represented by the BCA *P. terrestris* grown in vitro in liquid medium YPD; in blue are the *P. terrestris* DEGs during dual interaction with the host *M. domestica*, while in yellow are the *P. terrestris* DEGs during tritrophic interaction with the fungus *P. expansum* and with the host *M. domestica*; in light brown DEGs in common. (b - d) GO enrichment analysis of the *P. terrestris* DEGs carried out according to three groups depicted in the Venn Diagrams reported in a and c. The source data for Supplementary Figure 5a and 5b are in Supplementary Data 1 and 2, respectively, and the source data for Supplementary Figure 5c and 5d are in Supplementary Data 6.

a

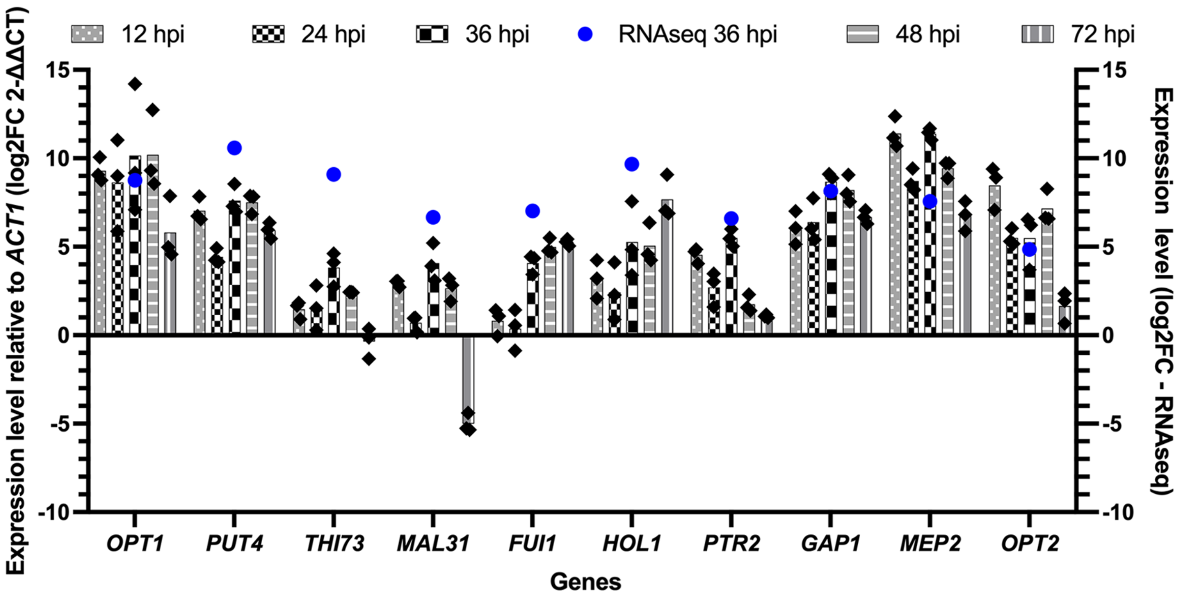

b

	Mean CT values obtained on untreated apple tisues using primers specific for PT22AV
ACT1	*35.5158*
TDH1	*21.9693*
OPT1	*32.4282*
PUT4	*35.7604*
THI73	*30.9260*
MAL31	*33.4446*
FUI1	*37.5365*
HOL1	*35.7940*
PTR2	*22.3929*
GAP1	*28.0806*
MEP2	*25.6626*
OPT2	*27.2596*

c

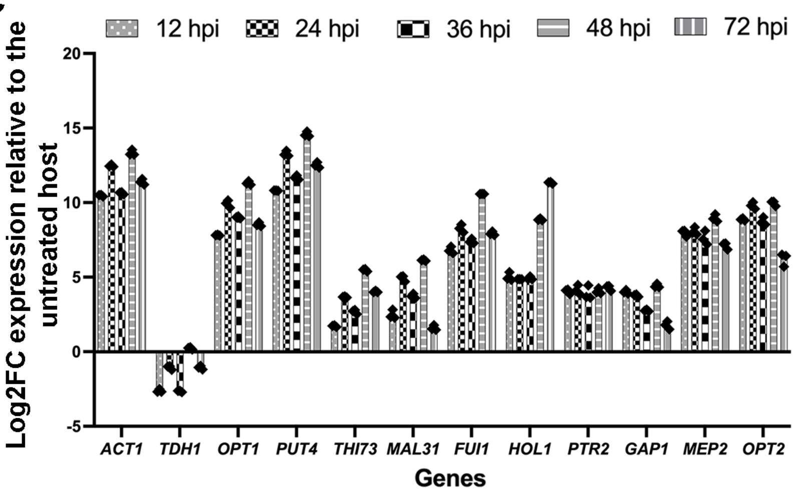

d

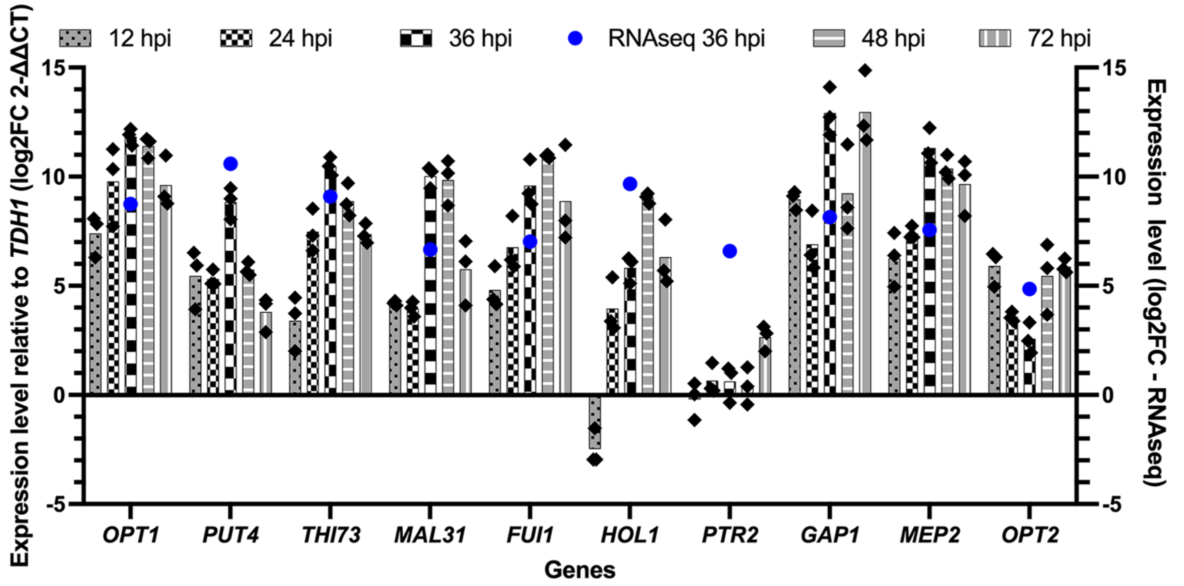

Supplementary Figure 6. Time course expression of selected genes of the BCA *P. terrestris*.

Real time qPCR of ten highly upregulated *P. terrestris* DEGs encoding predicted nutrients transporters identified through RNAseq analysis. Expression levels were analyzed at 12, 24, 36, 48 and 72 hpi from apple wounds (a) and apple medium (d) inoculated with the BCA *P. terrestris* PT22AV. In both cases the cDNA samples were standardized using the BCA *P. terrestris* incubated in liquid medium YPD for 12, 24, 36, 48 and 72 hours, and normalized with the actin-encoding gene *ACT1* (a) or the glyceraldehyde-3-phosphate dehydrogenase (GAPDH)-encoding gene *TDH1* (d). Data represent the mean of three technical replicates (n = 3).
In b and c is shown the interference of the apple cDNA with the primers specific for *P. terrestris* genes. (b) Mean CT values of three technical replicates obtained in qPCR reactions carried out using primers designed for *P. terrestris* genes. (c) Log2 fold change (FC) expression of the *P. terrestris* genes in the *in vivo* samples 'BCA in apple wounds' relative to the untreated host (2-ΔCT approach). Data represent the mean of three technical replicates (n = 3). A positive value means that the expression of the gene in the treated sample ('BCA in apple wounds') is higher than the expression of the same gene in the untreated host. The housekeeping gene *TDH1* was the only one that showed negative values, which means that its expression was higher in the untreated host and therefore it cannot be used as reference gene using the ΔΔCt formula. The source data for Supplementary Figure 6 is available in Supplementary Data 20.

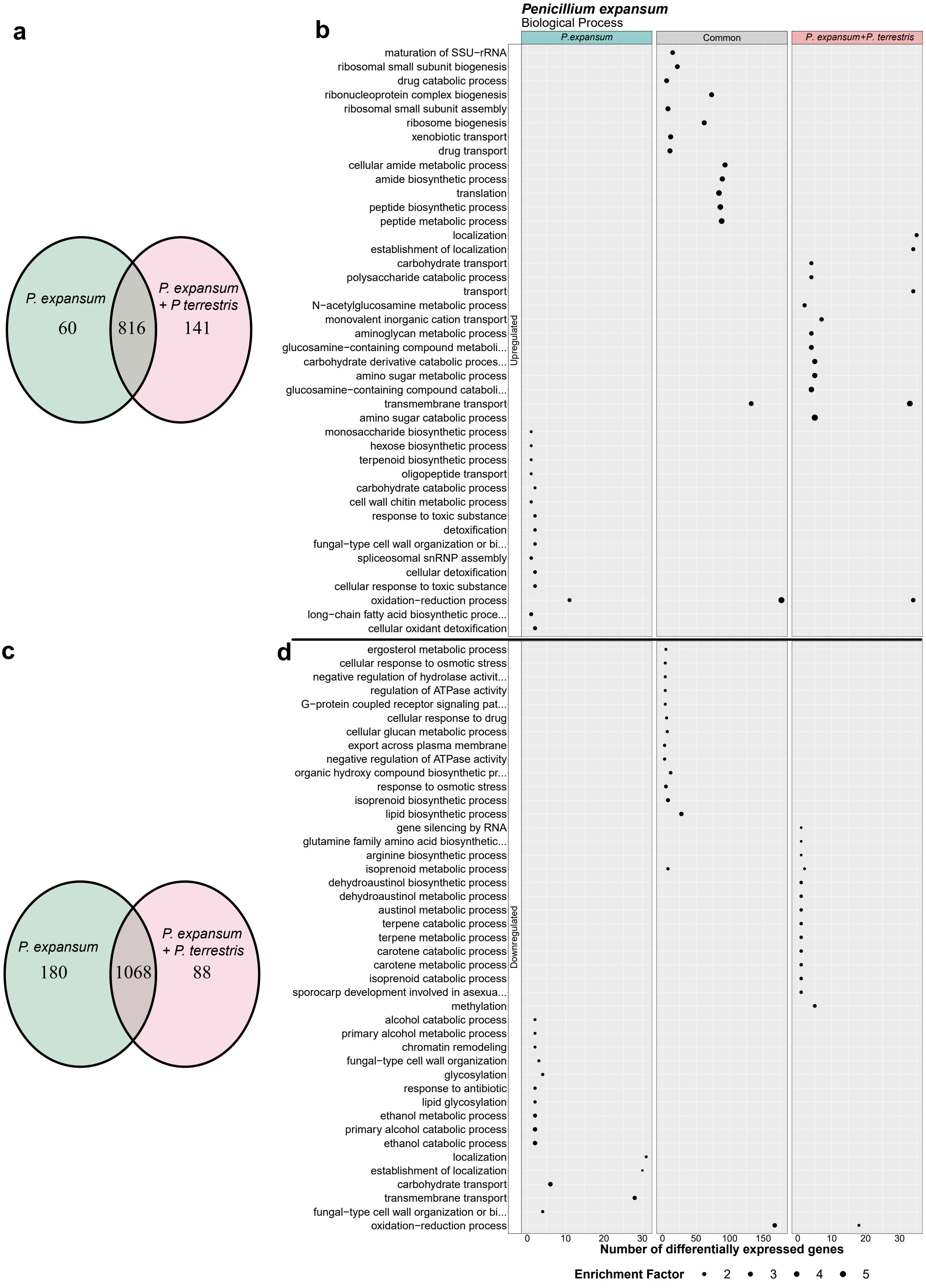
a
P. expansum
P. expansum
+ P terrestris
60
816
141
b
Penicillium expansum
Biological Process
P.expansum
Common
P. expansum+P. terrestris
Upregulated
maturation of SSU−rRNA
ribosomal small subunit biogenesis
drug catabolic process
ribonucleoprotein complex biogenesis
ribosomal small subunit assembly
ribosome biogenesis
xenobiotic transport
drug transport
cellular amide metabolic process
amide biosynthetic process
translation
peptide biosynthetic process
peptide metabolic process
localization
establishment of localization
carbohydrate transport
polysaccharide catabolic process
transport
N−acetylglucosamine metabolic process
monovalent inorganic cation transport
aminoglycan metabolic process
glucosamine−containing compound metaboli...
carbohydrate derivative catabolic proces...
amino sugar metabolic process
glucosamine−containing compound cataboli...
transmembrane transport
amino sugar catabolic process
monosaccharide biosynthetic process
hexose biosynthetic process
terpenoid biosynthetic process
oligopeptide transport
carbohydrate catabolic process
cell wall chitin metabolic process
response to toxic substance
detoxification
fungal−type cell wall organization or bi...
spliceosomal snRNP assembly
cellular detoxification
cellular response to toxic substance
oxidation−reduction process
long−chain fatty acid biosynthetic proce...
cellular oxidant detoxification
c
P. expansum
P. expansum
+ P. terrestris
180
1068
88
d
Downregulated
ergosterol metabolic process
cellular response to osmotic stress
negative regulation of hydrolase activit...
regulation of ATPase activity
G−protein coupled receptor signaling pat...
cellular response to drug
cellular glucan metabolic process
export across plasma membrane
negative regulation of ATPase activity
organic hydroxy compound biosynthetic pr...
response to osmotic stress
isoprenoid biosynthetic process
lipid biosynthetic process
gene silencing by RNA
glutamine family amino acid biosynthetic...
arginine biosynthetic process
isoprenoid metabolic process
dehydroaustinol biosynthetic process
dehydroaustinol metabolic process
austinol metabolic process
terpene catabolic process
terpene metabolic process
carotene catabolic process
carotene metabolic process
isoprenoid catabolic process
sporocarp development involved in asexua...
methylation
alcohol catabolic process
primary alcohol metabolic process
chromatin remodeling
fungal−type cell wall organization
glycosylation
response to antibiotic
lipid glycosylation
ethanol metabolic process
primary alcohol catabolic process
ethanol catabolic process
localization
establishment of localization
carbohydrate transport
transmembrane transport
fungal−type cell wall organization or bi...
oxidation−reduction process
0 10 20 30
0 50 100 150
0 10 20 30
Number of differentially expressed genes
Enrichment Factor 2 3 4 5

Supplementary Figure 7. Gene ontology analysis relative to the RNAseq analysis of the fungal pathogen *P. expansum*.

(a - c) Venn diagrams showing common and unique sets of upregulated (a) and downregulated (c) genes of the fungus *P. expansum* during dual and tritrophic interaction with the host *M. domestica* in the absence and in the presence of BCA *P. terrestris,* respectively; the comparative control condition is represented by the fungus *P. expansum* grown in vitro in liquid medium PDB; in green are the *P. expansum* DEGs during dual interaction with the host *M. domestica*, while in pink are the *P. expansum* DEGs during tritrophic interaction with the BCA *P. terrestris* and with the host *M. domestica*; in grey DEGs in common. (b - d) GO enrichment analysis of the *P. expansum* DEGs carried out according to three groups depicted in the Venn Diagrams reported in a and c. The source data for Supplementary Figure 7a and 7b are in Supplementary Data 8 and 9, respectively, and the source data for Supplementary Figure 7c and 7d are in Supplementary Data 12.

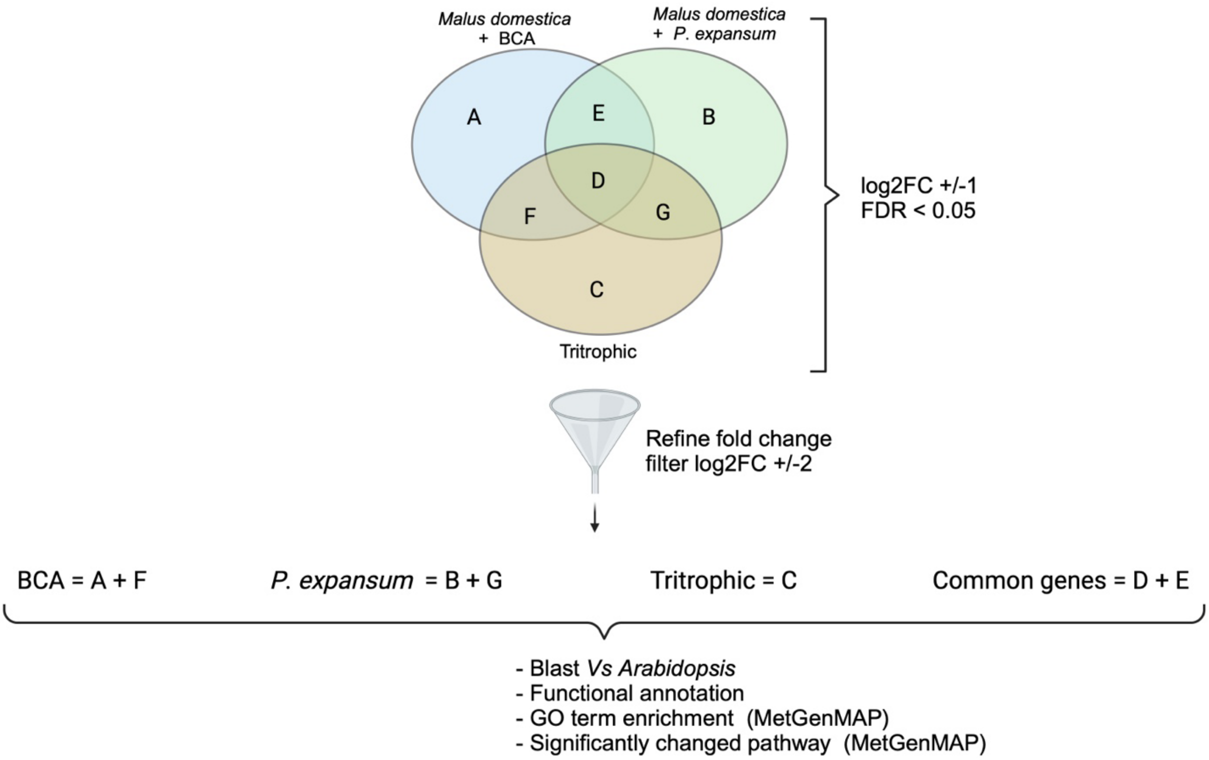

Supplementary Figure 8. Schematic representation of the pipeline used to obtain the final list of DEGs for *M. domestica*.

As a first step, all genes with a FDR <0.05 and with a log2FC +/- 1 were selected; subsequently, the data were compared with each other using the package VennDiagram, resulting in an additional datasets that include genes in common between the three conditions of study. As a last step, a further filtering step was carried out to select as DEGs those having a log2FC +/- 2. The function of the *M. domestica* DEGs was inferred by BLASTp against *Arabidopsis thaliana* genome (https://www.arabidopsis.org), and by GO and KEGG pathway enrichment. This figure was created with BioRender.com. The figure was exported on a paid subscription.

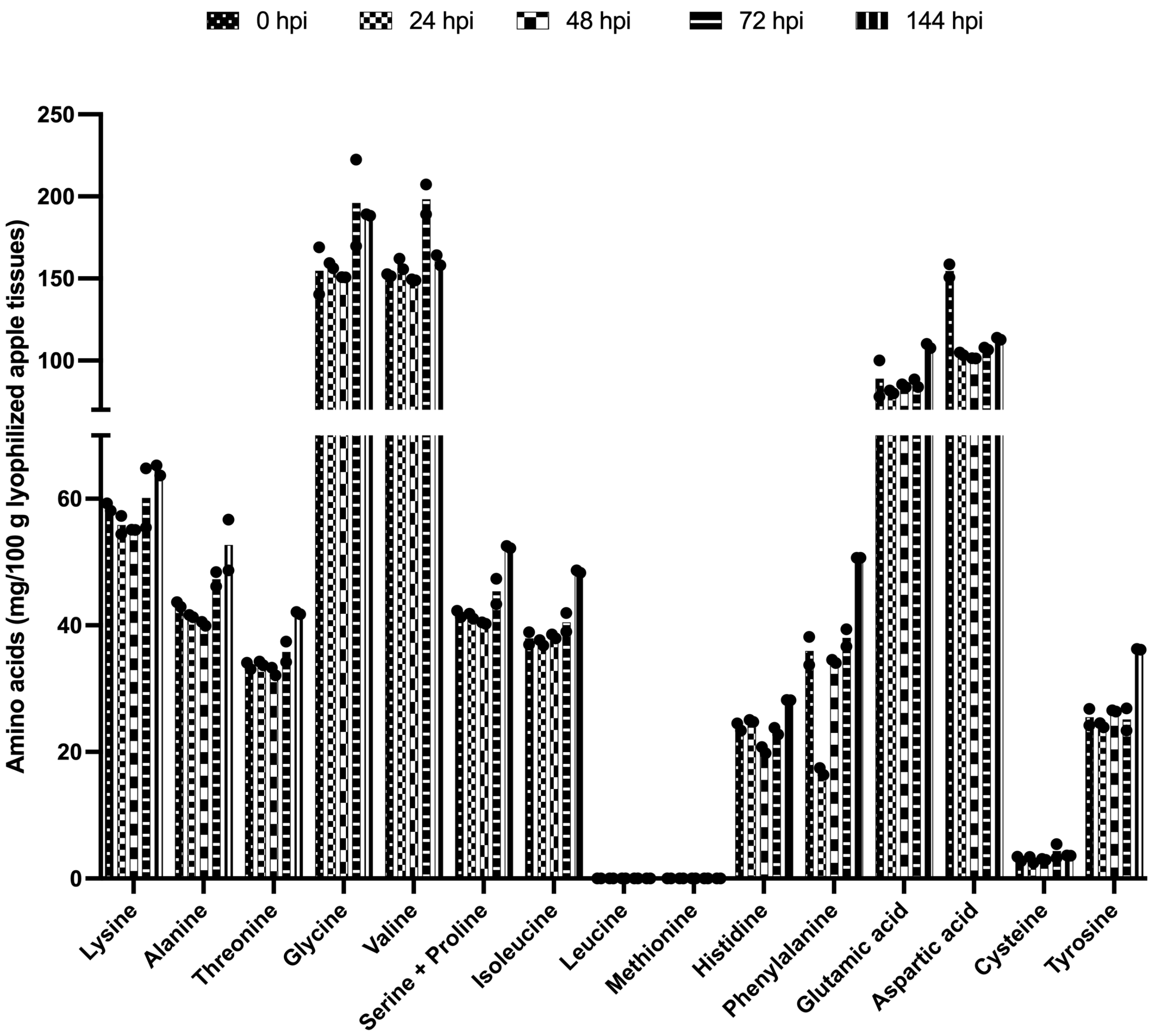

Supplementary Figure 9: amino acids analysis of apple wounds untreated and treated with the BCA *P. terrestris.*

Amino acids content of apple wounds untreated and treated with the BCA *P. terrestris* after 24, 48, 72 and 144 hpi; data are expressed as mg/100 mg dried apple tissues and represent the mean of two independent measurements (n = 2 biologically independent samples). The source data for Supplementary Figure 9 is available in Supplementary Data 20.

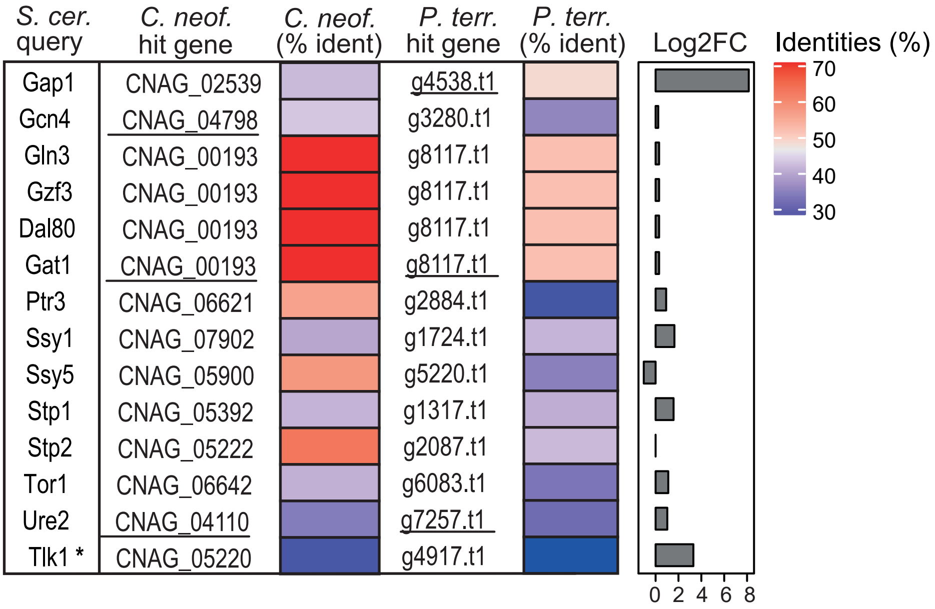

Supplementary Figure 10. Graphical representation of the conservation of the *S. cerevisiae* nitrogen regulators with the basidiomycetes *C. neoformans* strain H99 and *P. terrestris* PT22AV. The *S. cerevisiae* proteins have been retrieved from SGD and used as query for blastp against the genomes of *C. neoformans* strain H99 and *P. terrestris* PT22AV, with the percentage of identity obtained that is displayed in a color-coded scale (on the right panel). The last column reports the expression value of the *P. terrestris* PT22AV genes obtained during the dual interaction with the host *M. domestica*. The best *C. neoformans* and *P. terrestris* hits, reported in the respective columns, have been used for blastp (reciprocal blast) against *S. cerevisiae* to predict protein conservation and function. If the results of the reciprocal blastp were congruent, we predicted a conserved function of the proteins that are displayed as underlined: *C. neoformans* Gcn4, *P. terrestris* Gap1, and Gat1 and Ure2 of both *C. neoformans* and *P. terrestris* are predicted to be conserved with *S. cerevisiae*. The asterisk indicates the *C. neoformans* Tlk1 protein that has been used as query against *P. terrestris* PT22AV.

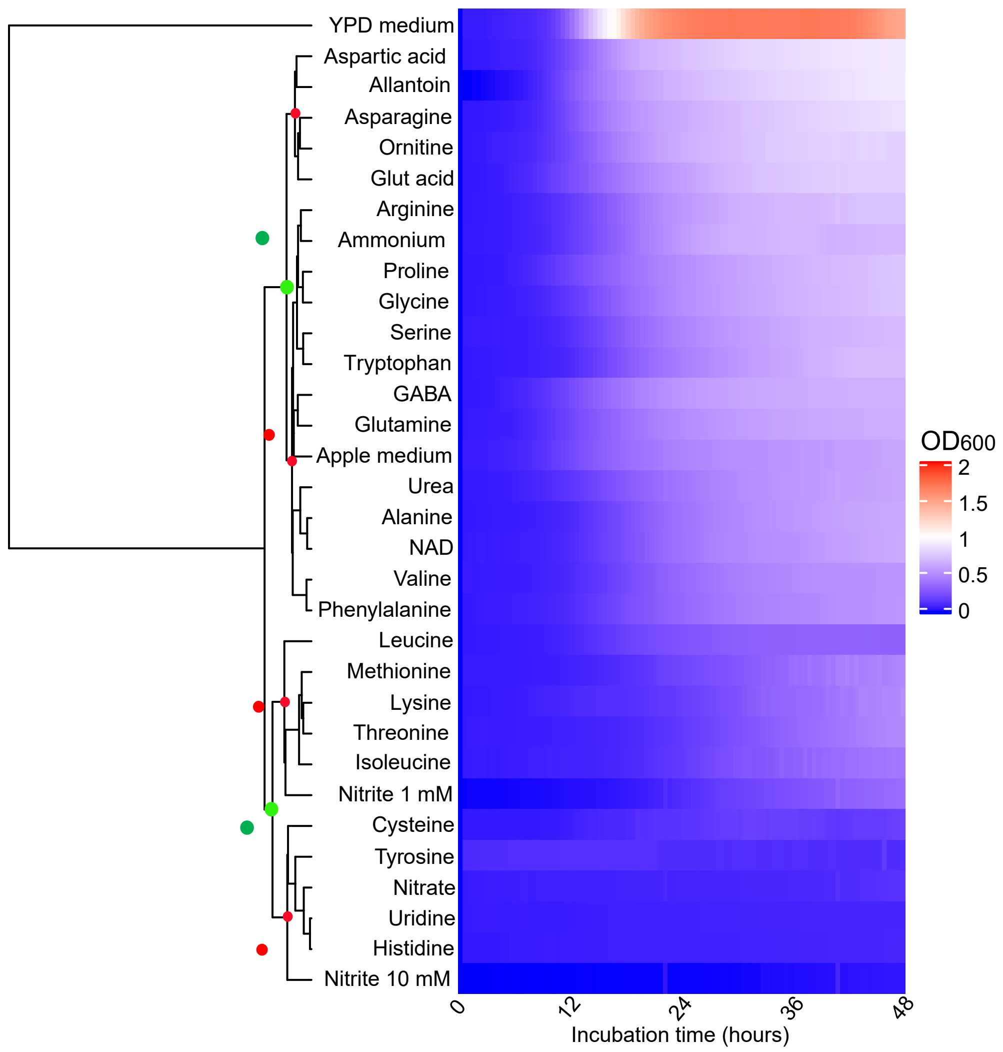

Supplementary Figure 11. Growth kinetics of *P. terrestris* PT22AV in several conditions. Growth kinetics of *P. terrestris* PT22AV in apple medium, and minimal medium supplemented with different nitrogen sources at 10 mM (with the exception of sodium nitrite used also at 1 mM). The green dots indicate the two main growth groups identified, good and poor growth; within each main group, two more groups were identified, as represented by red dots. The outgroup is represented by the growth kinetics of *P. terrestris* PT22AV in rich media YPD. The source data for Supplementary Figure 11 is available in Supplementary Data 20.

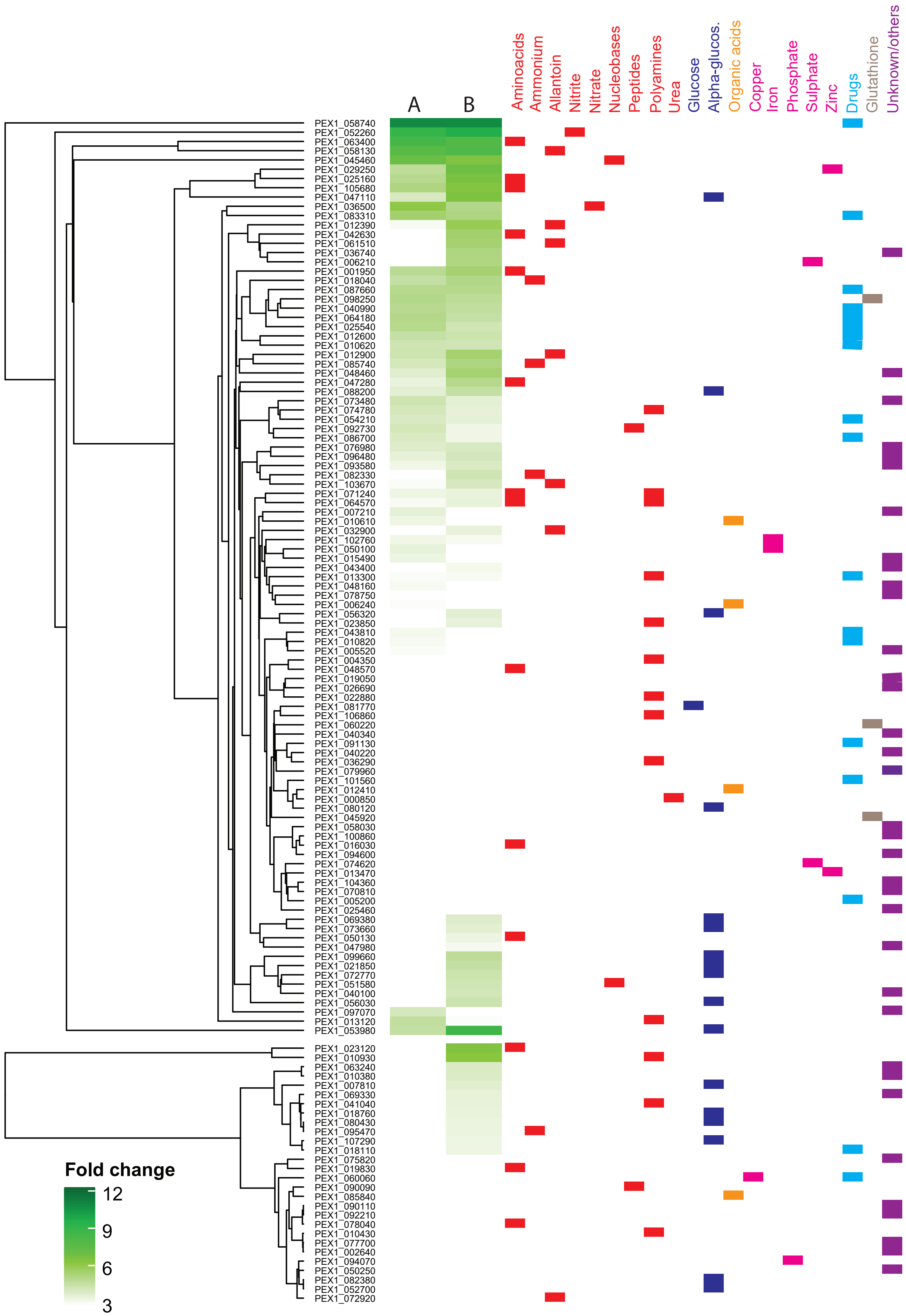

Supplementary Figure 12: Expression and function of the *P. expansum* DEGs-encoding for transporters.

On the left panels are represented heatmap and clustering analyses based on the gene expression changes, reported as log2FC, of the DEGs-encoding transporters of *P. expansum* during dual interaction with the host *M. domestica* (A), and during tritrophic interaction with the BCA *P. terrestris* and host *M. domestica* (B). On the right it is represented the predicted function of the *P. terrestris* DEGs-encoding transporters of nitrogen sources (red), sugars (blue), organic acids (orange), microelements (fuchsia), glutathione (grey), and drugs (light blue); in purple are represented transporters with unknown function or underrepresented group (eg only one DEG). The predicted function of the DEGs-encoding transporters is based on comparison with *S. cerevisiae* orthologs were possible, with the existing information of other fungal orthologs, and with the *P. expansum* genome annotation available [2]. The source data for Supplementary Figure 12 is available in Supplementary Data 20.

**Sample #replicate**	**Mapped against the genome of**	**Reads that mapped to one location (%)**	**Reads that mapped to multiple locations (%)**	**Unmapped reads (%)**
M. domestica #1	*M. domestica*	70.24	20.06	9.64
M. domestica #2	*M. domestica*	64.94	25.95	9.04
M. domestica + *P. expansum* #1	*M. domestica*	73.77	11.74	14.44
	P. expansum	8.53	0.01	91.46
M. domestica + *P. expansum* #2	*M. domestica*	71.45	8.05	20.45
	P. expansum	14.97	0.02	85.01
M. domestica + *P. expansum* #3	*M. domestica*	78.43	6.77	14.73
	P. expansum	7.81	0.01	92.17
M. domestica + *P. terrestris* #1	*M. domestica*	82.28	4.21	13.42
	P. terrestris	9.85	0.02	90.12
M. domestica + *P. terrestris* #2	*M. domestica*	73.74	7.93	18.26
	P. terrestris	12.71	0.04	87.24
M. domestica + *P. terrestris* #3	*M. domestica*	82.92	4.75	12.24
	P. terrestris	8.04	0.02	91.93
M. domestica + *P. terrestris* + *P. expansum* #1	*M. domestica*	74.77	4.36	20.80
	P. expansum	1.29	0.00	98.70
	P. terrestris	14.44	0.02	85.53
M. domestica + *P. terrestris* + *P. expansum* #2	*M. domestica*	68.32	4.70	26.92
	P. expansum	2.14	0.00	97.85
	P. terrestris	17.60	0.03	82.36
M. domestica + *P. terrestris* + *P. expansum* #3	*M. domestica*	76.84	4.18	18.88
	P. expansum	1.24	0.00	98.76
	P. terrestris	11.46	0.02	88.51
P. terrestris #1	*P. terrestris*	95.49	0.07	4.42
P. terrestris #2	*P. terrestris*	96.54	0.07	3.38
P. expansum #1	*P. expansum*	52.44	0.12	47.44
P. expansum #2	*P. expansum*	93.76	0.16	6.08
P. expansum #3	*P. expansum*	92.72	0.16	7.12

Supplementary Table 1: Mapping results of the samples used for RNAseq analysis. The hash symbol (#) indicates the number of the replicate for that sample.

Name	Sequence 5' - 3'	Gene	Gene name and usage
PVCB286	CGCCAACACGACGCAATATTCC	g5541.t1	*OPT1* qPCR F
PVCB287	GGTGTCCATCGCTGCTTG	g5541.t1	*OPT1* qPCR R
PVCB288	CGTTGGTGAGGTAGACAAT	g1105.t1	*PUT4* qPCR F
PVCB289	CCGTGTTCAACTGGATCGTC	g1105.t1	*PUT4* qPCR R
PVCB290	GAGGAAGTAGCGGATGGTCA	g4159.t1	*THI73* qPCR F
PVCB291	TGCTGATCGGGTACTGCGT	g4159.t1	*THI73* qPCR R
PVCB292	TGGAACTGGCGAGGTAGAG	g7507.t1	*MAL31* qPCR F
PVCB293	TGCAACTCGTGATTCTCCAC	g7507.t1	*MAL31* qPCR R
PVCB294	GCCGGATTCCTGAACAACAT	g1251.t1	*FUI1* qPCR F
PVCB295	CTCCGGGGTCCACTCGTA	g1251.t1	*FUI1* qPCR R
PVCB296	CGTAACCGAAGCCAAATGTCA	g4160.t1	*HOL1* qPCR F
PVCB297	CGTCCTCGTCTTGACCTAC	g4160.t1	*HOL1* qPCR R
PVCB298	AGTTGGGCCTTGTAGTTGGG	g1770.t1	*PTR2* qPCR F
PVCB299	TCTGTCTGTTCAACTCGGCC	g1770.t1	*PTR2* qPCR R
PVCB300	GCCCAAGATGTTCACGTACG	g4538.t1	*GAP1* qPCR F
PVCB301	GAGTGGCCCTGTATCTTCCA	g4538.t1	*GAP1* qPCR R
PVCB302	TTTCGTGTTGGGCAGAGTTG	g5563.t1	*MEP2* qPCR F
PVCB303	ATCCTGCACTTCATCCCCG	g5563.t1	*MEP2* qPCR R
PVCB304	CGGTGCTCCGTACAACTTGT	g5468.t1	*OPT2* qPCR F
PVCB305	GTACTGGACCGTGTTGCC	g5468.t1	*OPT2* qPCR R
PVCB312	CATTCGGTCGGCGATACCGTT	g7592.t1	*ACT1* qPCR F
PVCB313	GGTAACGAGCGATTCAGGT	g7592.t1	*ACT1* qPCR R
PVCB314	GTCATCCCTAGCCTCAACGG	g7373.t1	*TDH1* qPCR F
PVCB315	AAGATGGACGACTCGGTGG	g7373.t1	*TDH1* qPCR R

Supplementary Table 2: primers used in the present study

Mobile Phase (0.250 mL/min)				Time/potential waveform		
**Time** (min)	**H2O** (%)	**NaOH 250 mM** (%)	**NaOAc 1M** (%)	**Time (s)**	**Potential (V)**	**Integration**
0.0	80	20	0	0.00	+ 0.13	
2.0	80	20	0	0.04	+ 0.13	
12.0	80	20	0	0.05	+ 0.28	
16.0	68	32	0	0.11	+ 0.28	start
24.0	36	24	40	0.12	+ 0.60	
40.0	36	24	40	0.41	+ 0.60	
40.1	20	80	0	0.42	+ 0.28	end
42.1	20	80	0	0.56	+ 0.28	
42.1	80	20	0	0.57	- 1.67	
62.0	80	20	0	0.58	- 1.67	
				0.59	+ 0.93	
				0.60	+ 0.13	

Supplementary Table 3: Eluent gradient and time/potential waveform used for the amino acids chromatographic separation.

Supplementary References

1. Bianchi, F., van't Klooster, J. S., Ruiz, S. J. & Poolman, B. Regulation of Amino Acid Transport in *Saccharomyces cerevisiae*. *Microbiology and Molecular Biology Reviews* **83**, (2019).
2. Ballester, A. R. *et al.* Genome, transcriptome, and functional analyses of *Penicillium expansum* provide new insights into secondary metabolism and pathogenicity. *Molecular Plant-Microbe Interactions* **28**, 232–248 (2015).
