# Supplementary material for: Transcriptomic investigation of the interaction between a biocontrol yeast, Papiliotrema terrestris strain PT22AV, and the postharvest fungal pathogen Penicillium expansum on apple
Source: Commun Biol. 2024 Mar 22;7:359. doi: 10.1038/s42003-024-06031-w (PMC10960036; doi:10.1038/s42003-024-06031-w)
Supplement: Supplementary file 3 — Description of Supplementary Materials [file 42003_2024_6031_MOESM3_ESM.docx]

**Description of Additional Supplementary Files**

**File name:** Supplementary Data 1

**Description:** RNAseq output relative to the upregulated genes of the BCA P. terrestris. Upregulated DEGs (Differentially expressed genes) of P. terrestris, defined as those having FDR < 0.05 and a fold change >2, during interaction with the host, and with the fungus P. expansum; each condition is reported in a different excel sheet. See diagram in Supplementary Figure 3 for the procedure used to obtain the list of DEGs reported. Each page includes the P. terrestris gene name, the log2FC values and the FDR values as obtained from DESeq; then, we added manually the gene description according to the P. terrestris genome annotation, and the S. cerevisiae best hit and annotation following BLASTp against the Saccharomyces genome database (SGD). Genes are sorted based on the Log2FC values. For the common group, the sorting has been done considering the Log2FC values of the P. terrestris DEGs obtained during tritrophic interaction. #N/A = not available.

**File name:** Supplementary Data 2

**Description:** Gene Ontology of the genes of the BCA P. terrestris identified through RNAseq. GO of the upregulated DEGs of P. terrestris reported in Supplementary Data 1. For each condition are reported the results of the GO classification according to the three main categories “biological process”, “molecular function”, and “cellular components”; each condition is reported in a different excel sheet. #N/A = not available.

**File name:** Supplementary Data 3

**Description:** KEGG of the genes of the BCA P. terrestris identified through RNAseq. KEGG of the upregulated DEGs of P. terrestris reported in Supplementary Data 1. Each condition is reported in a different excel sheet.

**File name:** Supplementary Data 4

**Description:** Summary table of the RNAseq relative to the BCA P. terrestris. DEGs of P. terrestris highly expressed (Log2FC >4) during interaction with the host alone and coinoculated with P. expansum (common dataset) extracted from Supplementary Data 1 and 2. DEGs were organized for GO groups and/or their function, and sorted for Log2FC. For column 2, the gene name of the first S. cerevisiae hit following BLASTp has been used; where the GO and SGD description were discordant, both were included. Hypothetical proteins with no detected domains have not been included in this Table and can be found in Supplementary Data 1 and 2

**File name:** Supplementary Data 5

**Description:** Blast analysis of the P. terrestris transporters. S. cerevisiae transporters were searched in P. terrestris genome. The best hit in P. terrestris was subjected to reciprocal BLASTp to define whether the two proteins are orthologs and expected to have a predicted conserved function.

**File name:** Supplementary Data 6

**Description:** RNAseq output relative to the downregulated genes of the BCA P. terrestris. Downregulated DEGs of P. terrestris, defined as those having FDR < 0.05 and a fold change <2, during interaction with the host, and with the fungus P. expansum; each condition is reported in a different excel sheet. See diagram in Supplementary Figure 3 for the procedure used to obtain the list of DEGs reported. Each page includes the P. terrestris gene name, the log2FC values and the FDR values as obtained from DESeq; then, we added manually the gene description according to the P. terrestris genome annotation, and the S. cerevisiae best hit and annotation following BLASTp against the Saccharomyces genome database (SGD). Genes are sorted based on the Log2FC values. For the common group, the sorting has been done considering the Log2FC values of the P. terrestris DEGs obtained during tritrophic interaction. #N/A = not available.

**File name:** Supplementary Data 7

**Description:** P. terrestris genes showing opposite expression in the RNAseq analysis datasets. P. terrestris DEGs upregulated during the dual interaction with the host and downregulated during the tritrophic interaction with the fungus P. expansum on the host M. domestica, or viceversa. #N/A = not available.

**File name:** Supplementary Data 8

**Description:** RNAseq output relative to the upregulated genes of the fungal pathogen P. expansum. Upregulated DEGs of P. expansum, defined as those having FDR < 0.05 and a fold change >2, during interaction with the host, and with the BCA P. terrestris; each condition is reported in a different excel sheet. See diagram in Additional figure 3 for the procedure used to obtain the list of DEGs reported. Each page includes the P. expansum gene locus name, the log2FC values and the FDR values as obtained from DESeq; then, we added manually the gene description according to the P. expansum genome annotation, the NCBI Genbank transcript id with the relative protein annotation, and the S. cerevisiae best hit and annotation following BLASTp against SGD. Genes are sorted based on the Log2FC values. For the common group, the sorting has been done considering the Log2FC values of the P. expansum DEGs obtained during tritrophic interaction. #N/A = not available.

**File name:** Supplementary Data 9

**Description:** Gene Ontology of the genes of the fungal pathogen P. expansum identified through RNAseq. GO of the upregulated DEGs of P. expansum reported in Supplementary Data 8. For each condition are reported the results of the GO classification according to the three main categories “biological process”, “molecular function”, and “cellular components”; each condition is reported in a different excel sheet. #N/A = not available.

**File name:** Supplementary Data 10

**Description:** KEGG of the genes of the fungal pathogen P. expansum identified through RNAseq. KEGG of the upregulated DEGs of P. expansum reported in Supplementary Data 8. Each condition is reported in a different excel sheet.

**File name:** Supplementary Data 11

**Description:** Summary table of the RNAseq relative to the fungal pathogen P. expansum. DEGs of P. expansum highly expressed (Log2FC >4) during interaction with the host alone and coinoculated with P. terrestris (common dataset) extracted from Supplementary Data 8 and 9. DEGs were organized for GO groups and/or their function, and sorted for Log2FC. Where the GO and NCBI description were discordant, both were included. The studies where the same genes were identified are indicated.

**File name:** Supplementary Data 12

**Description:** RNAseq output relative to the downregulated genes of the fungal pathogen P. expansum. Downregulated DEGs of P. expansum, defined as those having FDR < 0.05 and a fold change <2, during interaction with the host, and with the BCA P. terrestris; each condition is reported in a different excel sheet. See diagram in Additional figure 3 for the procedure used to obtain the list of DEGs reported. Each page includes the P. expansum gene locus name, the log2FC values and the FDR values as obtained from DESeq; then, we added manually the gene description according to the P. expansum genome annotation, the NCBI Genbank transcript id with the relative protein annotation, and the S. cerevisiae best hit and annotation following BLASTp against the SGD. Genes are sorted based on the Log2FC values. For the common group, the sorting has been done considering the Log2FC values of the P. terrestris DEGs obtained during tritrophic interaction. #N/A = not available.

**File name:** Supplementary Data 13

**Description:** P. expansum genes showing opposite expression in the RNAseq analysis datasets. P. expansum DEGs upregulated during the dual interaction with the host and downregulated during the tritrophic interaction with the BCA P. terrestris on the host M. domestica, or viceversa. #N/A = not available.

**File name:** Supplementary Data 14

**Description:** RNAseq output relative to the upregulated genes of the host M. domestica. Upregulated DEGs of M. domestica, defined as those having FDR < 0.05 and a fold change >2, during interaction with the host, and with the BCA P. terrestris; each condition is reported in a different excel sheet and it refers to the grouping obtained through the Venn diagram of Figure 5b. See diagram in Supplementary Figure 8 for the procedure used to obtain the list of DEGs reported. Each page includes the M. domestica gene locus name, the log2FC values and the FDR values as obtained from DESeq; then, we added manually the gene description according to the M. domestica genome annotation, and the A. thaliana best hit and annotation following BLASTp against the TAIR (The Arabidopsis Information Resource). Genes are sorted based on the Log2FC values. For the common group, the sorting has been done considering the Log2FC values of the M. domestica DEGs obtained during interaction with the BCA and the fungus. #N/A = not available.

**File name:** Supplementary Data 15

**Description:** Gene Ontology of the upregulated genes of the host M. domestica. GO enrichment analysis of the upregulated DEGs of M. domestica reported in Supplementary Data 14; each condition is reported in a different excel sheet. #N/A = not available

**File name:** Supplementary Data 16

**Description:** Pathway analysis of the upregulated genes of the host M. domestica. Enrichment pathway analysis of the upregulated DEGs of M. domestica reported in Supplementary Data 14; each condition is reported in a different excel sheet. #N/A = not available.

**File name:** Supplementary Data 17

**Description:** RNAseq output relative to the downregulated genes of the host M. domestica. Downregulated DEGs of M. domestica, defined as those having FDR < 0.05 and a fold change <2, during interaction with the host, and with the BCA P. terrestris; each condition is reported in a different excel sheet and it refers to the grouping obtained through the Venn diagram of Figure 5c. See diagram in Supplementary Figure 8 for the procedure used to obtain the list of DEGs reported. Each page includes the M. domestica gene locus name, the log2FC values and the FDR values as obtained from DESeq; then, we added manually the gene description according to the M. domestica genome annotation, and the A. thaliana best hit and annotation following BLASTp against the TAIR (The Arabidopsis Information Resource). Genes are sorted based on the Log2FC values. For the common group, the sorting has been done considering the Log2FC values of the M. domestica DEGs obtained during interaction with P. terrestris. #N/A = not available

**File name:** Supplementary Data 18

**Description:** Gene Ontology of the downregulated genes of the host M. domestica. GO enrichment analysis of the downregulated DEGs of M. domestica reported in Table S8; each condition is reported in a different excel sheet. #N/A = not available

**File name:** Supplementary Data 19

**Description:** Pathway analysis of the downregulated genes of the host M. domestica. Enrichment pathway analysis of the downregulated DEGs of M. domestica reported in Table S11; each condition is reported in a different excel sheet. #N/A = not available.

**File name:** Supplementary Data 20

**Description:** Numerical source data for the figures and charts. Numerical source data for the Figures 2, 4 and 6, and for Supplementary Figures 6, 9, 11 and 12. The numerical source data for the remining Figures and Supplementary Figures are included in other Supplementary Data and they are specified in the legend of the items of interest.
