# Supplementary material for: Transcriptomic investigation of the interaction between a biocontrol yeast, Papiliotrema terrestris strain PT22AV, and the postharvest fungal pathogen Penicillium expansum on apple
Source: Commun Biol. 2024 Mar 22;7:359. doi: 10.1038/s42003-024-06031-w (PMC10960036; doi:10.1038/s42003-024-06031-w)
Supplement: Supplementary file 4 — Supplementary Data 1-20 [file 42003_2024_6031_MOESM4_ESM.zip › Supplementary Data 4.docx]

| ***P. terrestris* gene** | ***Saccharomyces cerevisiae* gene name** | **Log_2_FC *P. terrestris + P. expansum* in apple** | **Log_2_FC *P. terrestris* in apple** | **GO Description and/or SGD-NCBI-manual annotation** |
| --- | --- | --- | --- | --- |
| Transmembrane transport | | | | |
| g5541.t1 | *OPT1* | 11.27 | 8.75 | Oligopeptide transporter |
| g1105.t1 | *PUT4* | 10.70 | 10.59 | General amino acid permease (GO). Specific proline permease (SGD) |
| g4159.t1 | *THI73* | 9.61 | 9.09 | Putative plasma membrane permease proposed to be involved in carboxylic acid uptake |
| g7507.t1 | *MAL31* | 9.42 | 6.66 | Maltose permease |
| g5540.t1 | N/A | 8.77 | 6.61 | Inosine monophosphate (IMP) cyclohydrolase |
| g1251.t1 | *FUI1* | 8.66 | 7.02 | High affinity uridine permease |
| g4160.t1 | *HOL1* | 8.55 | 9.67 | MFS general substrate transporter |
| g7495.t1 | N/A | 8.30 | 9.19 | Formate nitrite transporter |
| g5118.t1 | N/A | 7.71 | 7.60 | MFS general substrate transporter |
| g1770.t1 | *PTR2* | 7.33 | 6.59 | Integral membrane peptide transporter |
| g2090.t1 | *AMF1* | 7.10 | 5.30 | MFS general substrate transporter (GO). Low affinity NH4+ transporter (SGD) |
| g840.t1 | *AMF1* | 6.90 | 3.98 | MFS general substrate transporter (GO). Low affinity NH4+ transporter (SGD) |
| g5936.t1 | *DUR3* | 6.82 | 7.59 | Transporter for urea and polyamines |
| g4538.t1 | *GAP1* | 6.77 | 8.15 | Amino acid transmembrane transporter |
| g5563.t1 | *MEP2* | 6.67 | 7.56 | Ammonium permease |
| g1860.t1 | *FLR1* | 6.21 | 4.63 | MFS general substrate transporter |
| g7238.t1 | *HNM1* | 6.16 | 6.72 | Transporter for choline. ethanolamine. and carnitine |
| g5890.t1 | *AGP2* | 6.10 | 6.33 | Amino acid transmembrane transporter (GO). Plasma membrane regulator of polyamine and carnitine transport (SGD) |
| g1180.t1 | *DIP5* | 6.07 | 5.25 | Amino acid transmembrane transporter (GO). Dicarboxylic amino acid permease (SGD) |
| g5468.t1 | *OPT2* | 5.90 | 4.85 | OPT oligopeptide transporter |
| g3316.t1 | *HXT2* | 5.81 | 10.43 | General substrate transporter (GO). High-affinity glucose transporter (SGD) |
| g1700.t1 | *MAL11* | 5.75 | 7.49 | High-affinity maltose transporter |
| g1990.t1 | *PUT4* | 5.60 | 6.47 | Amino acid transporter LysP (GO). Specific proline permease (SGD) |
| g5243.t1 | *SNF3* | 5.58 | 6.72 | Plasma membrane low glucose sensor |
| g7895.t1 | *DAL4* | 5.57 | 6.69 | Cytosine-purine permease (GO). Allantoin permease (SGD) |
| g1078.t1 | *DAL5* | 5.49 | 4.53 | MFS general substrate transporter (GO). Allantoate permease (SGD) |
| g2105.t1 | *THI73* | 5.47 | 7.23 | Putative plasma membrane permease proposed to be involved in carboxylic acid uptake |
| g5249.t1 | *MAL11* | 5.39 | 6.83 | High-affinity maltose transporter |
| g6201.t1 | N/A | 5.20 | 6.96 | 2-oxoglutarate-dependent ethylene/succinate-forming enzyme |
| g2664.t1 | *ITR2* | 5.15 | 8.12 | MFS general substrate transporter (GO). Myo-inositol transporter (SGD) |
| g5672.t1 | N/A | 5.11 | 6.39 | Chromate transmembrane transporter |
| g2284.t1 | N/A | 5.10 | 5.11 | Inner membrane transport protein yeiJ |
| g8082.t1 | *FLR1* | 5.05 | 3.71 | MFS general substrate transporter |
| g5688.t1 | *DAL5* | 4.99 | 5.59 | MFS general substrate transporter (GO). Allantoate permease (SGD) |
| g5745.t1 | *TPO3* | 4.92 | 4.80 | MFS general substrate transporter (GO). Polyamine transporter |
| g7415.t1 | *CPS1* | 4.90 | 3.79 | Zn-dependent exopeptidase (GO). Vacuolar carboxypeptidase S (SGD) |
| g3186.t1 | N/A | 4.83 | 4.58 | MFS general substrate transporter |
| g5485.t1 | *TNA1* | 4.53 | 4.97 | MFS general substrate transporter (GO). High affinity nicotinic acid permease (SGD) |
| g2442.t1 | N/A | 4.53 | 5.96 | Uric acid-xanthine permease |
| g8313.t1 | *JEN1* | 4.52 | 8.01 | MFS general substrate transporter (GO). Monocarboxylate/proton symporter (SGD) |
| g4109.t1 | *SEO1* | 4.48 | 5.97 | Major facilitator superfamily domain-containing protein |
| g5778.t1 | *DAL4* | 4.47 | 4.45 | Uridine permease/thiamine transporter/allantoin transport (GO). Allantoin permease (SGD) |
| g826.t1 | *OPT1* | 4.47 | 4.31 | OPT family small oligopeptide transporter |
| g6361.t1 | YIL166C | 4.44 | 6.02 | MFS general substrate transporter |
| g2154.t1 | *GLO4* | 4.30 | 4.35 | Mitochondrial glyoxalase II |
| g6693.t1 | *MAL31* | 4.27 | 4.61 | Substrate-specific transmembrane transporter (GO). Maltose permease |
| g2622.t1 | *DUR3* | 4.20 | 6.66 | Urea active transporter |
| g2623.t1 | *DUR3* | 4.19 | 7.05 | SSS family solute:Na+ symporter (GO). Plasma membrane transporter for both urea and polyamines (SGD) |
| g4161.t1 | N/A | 4.13 | 4.67 | N-substituted formamide deformylase |
| g7813.t1 | N/A | 4.11 | 6.05 | Chromate transmembrane transporter |
| g7316.t1 | *AMF1* | 4.07 | 1.44 | MFS general substrate transporter (GO). Low affinity NH4+ transporter (SGD) |
| g2297.t1 | *FET5* | 4.05 | 2.65 | Multicopper oxidases |
| g825.t1 | *OPT1* | 4.05 | 4.63 | OPT family small oligopeptide transporter |
| g5716.t1 | *TPO5* | 4.04 | 2.82 | Amino acid transporter (GO). Putative polyamine transporter (SGD) |
| g7969.t1 | YIL166C | 4.04 | 6.25 | MFS general substrate transporter |
| Oxidation-reduction process | | | | |
| g3330.t1 | N/A | 10.38 | 7.50 | Nucleoside-diphosphate-sugar epimerase |
| g6692.t1 | N/A | 9.69 | 7.57 | NADP(+)-dependent serine dehydrogenase and carbonyl reductase |
| g6691.t1 | N/A | 9.53 | 7.37 | Short-chain dehydrogenase/reductase like protein yusS |
| g5806.t1 | *AYR1* | 9.06 | 5.49 | Bifunctional triacylglycerol lipase and 1-acyl DHAP phosphate reductase involved in phosphatidic acid biosynthesis |
| g4444.t1 | *GOR1* | 7.77 | 8.79 | Glyoxylate reductase |
| g3727.t1 | *ENV9* | 7.61 | 7.12 | Protein proposed to be involved in vacuolar functions |
| g6644.t1 | *GAL7* | 7.59 | 6.55 | Galactose-1-phosphate uridyl transferase |
| g2444.t1 | N/A | 7.52 | 5.40 | Flavin containing amine oxidoreductase |
| g5935.t1 | *AMD2* | 7.10 | 7.92 | Putative amidase |
| g7494.t1 | NA | 7.00 | 7.63 | Nitrite reductase NiiA |
| g4212.t1 | NA | 6.73 | 6.98 | Putative zinc-binding oxidoreductase ToxD involved in copper metabolism |
| g1952.t1 | *EXG1* | 6.48 | 8.46 | Major exo-1.3-beta-glucanase of the cell wall |
| g1043.t1 | N/A | 6.24 | 3.00 | Intradiol ring-cleavage dioxygenase |
| g3941.t1 | YEL023C | 6.15 | 6.81 | Putative short chain dehydrogenase reductase |
| g5317.t1 | *GAL1* | 6.14 | 5.23 | Galactokinase |
| g1673.t1 | NA | 6.08 | 4.87 | NADP-binding rossmann-fold containing |
| g2435.t1 | *GOR1* | 6.05 | 5.95 | D-specific alpha-keto acid dehydrogenase (GO). Glyoxylate reductase (SGD) |
| g137.t1 | *ICL1* | 5.73 | 6.15 | Isocitrate lyase that catalyzes the formation of succinate and glyoxylate from isocitrate (glyoxylate cycle) |
| g1870.t1 | *KIN3* | 5.73 | 6.66 | Calcium/calmodulin-dependent protein kinase (GO). Serine/threonine protein kinase (SGD) |
| g6407.t1 | *FOX2* | 5.49 | 5.10 | 3-hydroxyacyl-CoA dehydrogenase and enoyl-CoA hydratase (peroxisomal fatty acid beta-oxidation pathway) |
| g4715.t1 | YLL056C | 5.43 | 3.31 | Putative NADH-flavin oxidoreductase |
| g730.t1 | N/A | 5.39 | 4.34 | Putative short chain dehydrogenase |
| g5796.t1 | N/A | 5.23 | 5.10 | Glutamyl-tRNA amidotransferase |
| g6694.t1 | N/A | 4.99 | 5.30 | Alpha-galactosidase C |
| g7491.t1 | N/A | 4.93 | 4.66 | FAD dependent oxidoreductase |
| g2051.t1 | YMR034C | 4.93 | 11.60 | Putative sodium/bile acid cotransporter 7-B/bile acid cotransporter 7-B |
| g3611.t1 | N/A | 4.82 | 4.39 | Myo-inositol oxygenase |
| g1175.t1 | N/A | 4.66 | 4.00 | Acidic repeat-containing protein |
| g258.t1 | *SER3* | 4.60 | 5.47 | D-isomer specific 2-hydroxyacid dehydrogenase (GO). 3-phosphoglycerate dehydrogenase (SGD) |
| g2744.t1 | N/A | 4.59 | 6.04 | N-methyl-L-tryptophan oxidase |
| g6121.t1 | *LYS9* | 4.50 | 5.57 | Saccharopine dehydrogenase (lysine biosynthesis) |
| g7289.t1 | N/A | 4.30 | 4.71 | NADPH-dependent FMN reductase ArsH |
| g1800.t1 | N/A | 4.25 | 6.41 | NADP-dependent mannitol dehydrogenase |
| g4708.t1 | *IRC24* | 4.20 | 6.73 | Putative benzil reductase similar to short-chain dehydrogenase/reductases |
| Glutamine family aminoacid metabolic process | | | | |
| g7506.t1 | *ARG4* | 10.37 | 8.01 | Argininosuccinate lyase |
| g6404.t1 | N/A | 4.70 | 4.19 | Glutamine synthetase guanido kinase |
| g1107.t1 | *UGA2* | 4.56 | 5.50 | Succinate semialdehyde dehydrogenase involved in the utilization of gamma-aminobutyrate (GABA) as a nitrogen source |
| g3457.t1 | N/A | 4.55 | 5.92 | Glycoside hydrolase |
| g2747.t1 | N/A | 4.42 | 2.81 | Proline dehydrogenase |
| g2450.t1 | ASP3-4 | 4.13 | 8.92 | L-asparaginase II |
| g4161.t1 | N/A | 4.12 | 4.67 | N-substituted formamide deformylase |
| Regulation of transcription by RNA polymerase II | | | | |
| g7286.t1 | *YRM1* | 4.17 | 4.09 | Zinc finger transcription factor involved in multidrug resistance |
| Unclassified GO | | | | |
| g3033.t1 | N/A | 12.21 | 10.31 | NAD(P)-binding Rossmann-fold domain |
| g4389.t1 | N/A | 11.48 | 14.16 | Transmembrane domains |
| g3623.t1 | N/A | 7.74 | 7.93 | Putative dehydrogenasis with a glycine-rich NAD(P)-binding motif |
| g7073.t1 | N/A | 7.73 | 6.02 | Glutathione-dependent formaldehyde-activating gfa protein |
| g1937.t1 | N/A | 6.50 | 6.16 | Marvel domain containing protein |
| g3542.t1 | N/A | 4.47 | 4.32 | Glutathione-dependent formaldehyde-activating enzyme/centromere protein V |
| g1926.t1 | N/A | 6.38 | 4.91 | Putative carboxypeptidase |
| g1250.t1 | N/A | 6.28 | 4.32 | Putative methyltransferase |
| g6237.t1 | *NIT1* | 5.80 | 6.19 | Aliphatic nitrilase |
| g7322.t1 | N/A | 5.58 | 4.65 | Lactonase |
| g5400 | *FSH2* | 5.37 | 6.75 | Putative serine hydrolase |
| g1699.t1 | N/A | 5.35 | 8.13 | Putative endoglucanase |
| g5568.t1 | N/A | 5.04 | 8.56 | Glycoside hydrolase family 79 protein |
| g6785.t1 | N/A | 4.25 | 4.81 | Serine/threonine protein kinase |
