# Supplementary material for: Transcriptomic investigation of the interaction between a biocontrol yeast, Papiliotrema terrestris strain PT22AV, and the postharvest fungal pathogen Penicillium expansum on apple
Source: Commun Biol. 2024 Mar 22;7:359. doi: 10.1038/s42003-024-06031-w (PMC10960036; doi:10.1038/s42003-024-06031-w)
Supplement: Supplementary file 4 — Supplementary Data 1-20 [file 42003_2024_6031_MOESM4_ESM.zip › Supplementary Data 5.docx]

| ***S. cerevisiae* gene name**  **(query)** | **Function** | ***P. terrestris* hit (Sc query)** | **Reciprocal BLASTp hit**  **(Pt query)** | **Conserved protein** |
| --- | --- | --- | --- | --- |
| 1 - Plasma membrane transporters | | | | |
| 1.1 YAT (Yeast Aminoacid Transporters) | | | | |
| *AGP1* | Broad-specificity, low-affinity amino acid permease; minor serine permease with major contributions from paralog Gnp1p; involved in uptake of asparagine, glutamine, and other amino acids | g4538 | *GAP1* | NO |
| *AGP2* | Plasma membrane regulator of polyamine and carnitine transport. | g5980 | *AGP2* | YES |
| *AGP3* | Low-affinity amino acid permease; may act to supply the cell with amino acids as nitrogen source in nitrogen-poor conditions | g4538 | *GAP1* | NO |
| *ALP1* | Basic amino acids; arginine transporter | g1724 | *DIP5* | NO |
| *BAP2* | High-affinity leucine permease; functions as a branched-chain amino acid permease involved in uptake of leucine, isoleucine and valine | g4538 | *GAP1* | NO |
| *BAP3* | Amino acid permease; involved in uptake of cysteine, leucine, isoleucine and valine | g4538 | *GAP1* | NO |
| *CAN1* | Arginine, lysine, ornithine and canavanine transporter | g1724 | *DIP5* | NO |
| *DIP5* | Dicarboxylic amino acids; mediates high-affinity and high-capacity transport of L-glutamate and L-aspartate; also a transporter for Gln, Asn, Ser, Ala, and Gly | g4710 | *DIP5* | YES |
| *GAP1* | General amino acid permease; involved in uptake of all L-amino acids | g4538 | *GAP1* | YES |
| *GNP1* | Broad specificity amino acid permease; high-affinity glutamine permease; major serine permease with minor contributions from paralog Agp1p; also transports Leu, Thr, Cys, Met and Asn | g4538 | *GAP1* | NO |
| *HIP1* | High-affinity histidine permease | g4538 | *GAP1* | NO |
| *MMP1* | S-MethylMethionine Permease | g4538 | *GAP1* | NO |
| *LYP1* | Lysine permease; one of three amino acid permeases (Alp1, Can1, Lyp1) responsible for uptake of cationic amino acids | g1724 | *DIP5* | NO |
| *PUT4* | Proline permease | g1724 | *DIP5* | NO |
| *SAM3* | S-adenosylmethionine (high-affinity) transporter | g4538 | *GAP1* | NO |
| *TAT1* | Amino acid transporter for valine, leucine, isoleucine, and tyrosine; low-affinity tryptophan and histidine transporter | g4538 | *GAP1* | NO |
| *TAT2* | High affinity tryptophan and tyrosine permease | g4538 | *GAP1* | NO |
| 1.2 APC (Amino acid-polyamine-organocation) | | | | |
| *MUP1* | High affinity methionine permease involved in both methionine and cysteine uptake | g1209 | *MUP1* | YES |
| *MUP3* | Low-affinity methionine permease | g1209 | *MUP1* | YES |
| 1.3 MFS (major facilitator superfamily) | | | | |
| *YCT1* | High-affinity cysteine-specific transporter | g2117 | *YCT1* | YES |
| *VBA5* | L-arginine transporter | g4931 | *VBA5* | YES |
| 2 Vacuolar transporters | | | | |
| 2.1 MFS (major facilitator superfamily) | | | | |
| *VBA1* | Histidine and lysine transporter | g8067 | *VBA1* | YES |
| *VBA2* | Histidine, lysine and arginine transporter | g1523 | *VBA2* | YES |
| *VBA3* | Histidine and lysine transporter | g3059 | *VBA5* | NO |
| *ATG22* | Isoleucine, Leucine and tyrosine transporter | g2774 | *ATG22* | YES |
| 2.2 AAAP (amino acid/auxin permease) | | | | |
| *AVT1* | Glutamine, isoleucine, tyrosine, and histidine transmembrane transporter | g6836 | *AVT1* | YES |
| *AVT3* | Glutamine, isoleucine, tyrosine transmembrane transporter | g3545 | *AVT3* | YES |
| *AVT4* | Glutamine, isoleucine, tyrosine, histidine, lysine and arginine transmembrane transporter | g3545 | *AVT3* | NO |
| *AVT6* | Aspartate and glutamate transmembrane transporter | g70 | *AVT6* | YES |
| *AVT7* | Proline and glutamine transmembrane transporter | g70 | *AVT6* | NO |
| 2.3 LCT (lysosomal cystine transporter) | | | | |
| *ERS1* | Cystine transmembrane transporter | g1825 | *ERS1* | YES |
| *YPQ1* | Lysine, Arginine transporter | g3207 | *RTC1 (YPQ1* paralog*)* | YES |
| *YPQ2* | Arginine transporter | g3207 | *RTC1 (YPQ1* paralog*)* | NO |
| *YPQ3* | Histidine transporter | g3207 | *RTC1 (YPQ1* paralog*)* | NO |
| 3 Mitochondrial transporters | | | | |
| 3.1 MC (mitochondrial carrier) | | | | |
| *AGC1* | Glutamate uniporter and as an aspartate-glutamate exchanger | g3200 | *AGC1* | YES |
| *HEM25* | Glycine transporter | g5497 | *HEM25* | YES |
| *ORT1* | Lysine, Arginine, ornithine transporter | g1358 | *ORT1* | YES |
| *PET8* | *S*-Adenosylmethionine, *S*-adenosylhomocysteine transporter | g1008 | *PET8* | YES |
| 4 Other | | | | |
| *UGA4* (APC) | High-affinity GABA (gamma-aminobutyrate) permease | g166 | *UGA4* | YES |
| *AQR1* (MFS) | Alanine, Glutamic acid, Asparagine transporter | g46 | *QDR2* | NO |
