# Supplementary material for: Transcriptomic investigation of the interaction between a biocontrol yeast, Papiliotrema terrestris strain PT22AV, and the postharvest fungal pathogen Penicillium expansum on apple
Source: Commun Biol. 2024 Mar 22;7:359. doi: 10.1038/s42003-024-06031-w (PMC10960036; doi:10.1038/s42003-024-06031-w)
Supplement: Supplementary file 4 — Supplementary Data 1-20 [file 42003_2024_6031_MOESM4_ESM.zip › Supplementary Data 11.docx]

| ***P. expansum* gene** | **LogFC *P. expansum*** | **LogFC *P. expansum* + *P. terrestris*** | **GO Description and/or SGD-NCBI-manual annotation** | **Studies where the gene has been identified** |
| --- | --- | --- | --- | --- |
| **Oxidation reduction process** | | | | |
| PEX1_083840 | 13.71 | 13.75 | UDP-glucuronosyl and UDP-glucosyl transferase |  |
| PEX1_010540 | 11.22 | 9.62 | Cytochrome b5 reductase Cbr1 |  |
| PEX1_023520 | 8.22 | 9.47 | Aromatic-ring-hydroxylating dioxygenase, alpha subunit | Ballester et al., 2015 (72 h) |
| PEX1_019820 | 5.86 | 8.63 | Monooxygenase of the alternative pyrimidine degradation pathway |  |
| PEX1_104640 | 6.97 | 8.51 | Retinal dehydrogenase | Ballester et al., 2015 (72 h) |
| PEX1_090500 | 6.24 | 8.40 | Metallo-dependent hydrolase |  |
| PEX1_019800 | 5.69 | 8.34 | Nitroreductase |  |
| PEX1_010530 | 10.29 | 8.31 | Cytochrome P450 monooxygenase Erg11 |  |
| PEX1_028050 | 7.59 | 8.21 | Proline dehydrogenase | Ballester et al., 2015 (48 h) |
| PEX1_003290 | 7.86 | 7.96 | Sulfide quinone-reductase |  |
| PEX1_088180 | 5.12 | 7.72 | Non-heme dioxygenase in morphine synthesis N-terminal |  |
| PEX1_010400 | 2.93 | 7.36 | Citrinin biosynthesis oxygenase CtnA |  |
| PEX1_012560 | 6.24 | 7.29 | Cellobiose dehydrogenase | Ballester et al., 2015 (72 h) |
| PEX1_103650 | 6.80 | 7.22 | FMN-dependent alpha-hydroxy acid dehydrogenase |  |
| PEX1_079720 | 7.59 | 7.19 | N,N-dimethylglycine oxidase | Ballester et al., 2015 (48 h) |
| PEX1_025200 | 5.86 | 7.10 | Ketopantoate reductase |  |
| PEX1_007490 | 6.00 | 6.90 | Glucose oxidase Gox2 involved in production of D-gluconic acid | Ballester et al., 2015 (48 – 72 h); Barad et al., 2012 |
| PEX1_081800 | 4.17 | 6.82 | Alcohol dehydrogenase superfamily, zinc-containing |  |
| PEX1_062750 | 6.78 | 6.82 | Linoleoyl-CoA desaturase |  |
| PEX1_021020 | 6.82 | 6.69 | Sterol 24-C-methyltransferase |  |
| PEX1_100010 | 6.68 | 6.67 | Oxidoreductase |  |
| PEX1_043330 | 3.38 | 6.46 | NADPH-dependent quinone reductase Zta1 |  |
| PEX1_003340 | 2.12 | 6.46 | Bifunctional solanapyrone synthase |  |
| PEX1_025190 | 4.86 | 6.43 | Phytanoyl-CoA dioxygenase |  |
| PEX1_051680 | 4.30 | 6.31 | 1,3-beta-glucanosyltransferase |  |
| PEX1_012510 | 3.92 | 6.26 | Stellatic acid synthase |  |
| PEX1_010510 | 5.27 | 5.99 | FAD linked oxidase, N-terminal |  |
| PEX1_052120 | 5.81 | 5.96 | Aromatic-ring-hydroxylating dioxygenase, alpha subunit | Ballester et al., 2015 (48 h) |
| PEX1_083320 | 6.64 | 5.96 | Benzoate 4-monooxygenase cytochrome P450 |  |
| PEX1_083590 | 5.28 | 5.90 | Phenol hydroxylase reductase |  |
| PEX1_074900 | 9.13 | 5.71 | Fumagillin dodecapentaenoate synthase |  |
| PEX1_067810 | 6.64 | 5.57 | Alpha-ketoglutarate dependent xanthine dioxygenase |  |
| PEX1_014840 | 2.96 | 5.53 | Short-chain dehydrogenase reductase sdr |  |
| PEX1_090100 | 3.93 | 5.42 | D-amino acid oxidase |  |
| PEX1_039520 | 3.19 | 5.54 | Related to nitrate reductase |  |
| PEX1_036480 | 5.68 | 5.33 | Nitrate reductase niaD |  |
| PEX1_103560 | 3.13 | 5.29 | ISP domain-containing protein |  |
| PEX1_023510 | 4.18 | 5.28 | Aromatic-ring-hydroxylating dioxygenase, alpha subunit | Ballester et al., 2015 (72 h) |
| PEX1_028060 | 4.62 | 5.14 | Delta-1-pyrroline-5-carboxylate dehydrogenase mitochondrial | Ballester et al., 2015 (48 h) |
| PEX1_088240 | 4.81 | 5.12 | FMN-dependent alpha-hydroxy acid dehydrogenase |  |
| PEX1_082120 | 3.76 | 5.03 | Alpha-ketoglutarate-dependent sulfonate dioxygenase |  |
| PEX1_048780 | 4.87 | 4.98 | Glutathione-dependent formaldehyde-activating enzyme (Fragment) |  |
| PEX1_071260 | 4.49 | 4.79 | Clavaminate synthase-like protein |  |
| PEX1_051180 | 3.49 | 4.77 | Benzoate 4-monooxygenase cytochrome | Barad et al., 2016 |
| PEX1_020170 | 2.45 | 4.74 | Reductase with broad range of substrate specificity |  |
| PEX1_040410 | 3.15 | 4.74 | Glucose-methanol-choline oxidoreductase |  |
| PEX1_083680 | 4.79 | 4.71 | Amidase signature enzyme | Ballester et al., 2015 (48 h) |
| PEX1_086320 | 4.19 | 4.65 | Aspartate-semialdehyde dehydrogenase |  |
| PEX1_011780 | 7.32 | 4.63 | Glucose oxidase Gox3 | Ballester et al., 2015 (24-48-72) |
| PEX1_060490 | 5.23 | 4.57 | Transketolase, C-terminal/Pyruvate-ferredoxin oxidoreductase, domain II |  |
| PEX1_025610 | 7.17 | 4.56 | Pyoverdine dityrosine biosynthesis |  |
| PEX1_073650 | 2.44 | 4.56 | Phytanoyl-CoA dioxygenase |  |
| PEX1_099520 | 3.64 | 4.55 | Primary-amine oxidase |  |
| PEX1_045780 | 6.71 | 4.54 | Extracellular rhamnogalacturonase |  |
| PEX1_036730 | 2.05 | 4.52 | Alpha-ketoglutarate dependent xanthine dioxygenase |  |
| PEX1_103680 | 3.06 | 4.47 | Non-heme dioxygenase N-terminal domain |  |
| PEX1_083580 | 3.08 | 4.31 | Cytochrome P450 monooxygenase |  |
| PEX1_023260 | 3.95 | 4.29 | Toxin biosynthesis ketoreductase |  |
| PEX1_002310 | 4.68 | 4.24 | Mitochondrial aldehyde dehydrogenase Ald4 | Ballester et al., 2015 (48 h) |
| PEX1_016620 | 5.09 | 4.23 | Prostaglandin dehydrogenase |  |
| PEX1_024470 | 3.69 | 4.16 | Pyrroline-5-carboxylate reductase dimerization | Ballester et al., 2015 (48 h) |
| PEX1_039380 | 4.22 | 4.15 | Cysteine dioxygenase type I | Barad et al., 2016 |
| PEX1_081840 | 4.06 | 4.09 | Catalase, mono-functional, heme-containing | Ballester et al., 2015 (48 h) |
| PEX1_049460 | 4.16 | 6.55 | Carbon-nitrogen ligase activity, with glutamine as amido-N-donor |  |
| PEX1_059870 | 6.35 | 5.82 | Formate dehydrogenase | Barad et al., 2016 |
| PEX1_080170 | 2.96 | 4.10 | Aminoglycoside phosphotransferase |  |
| **Transport** | | | | |
| PEX1_058740 | 10.72 | 10.67 | Putative drug/proton antiporter |  |
| PEX1_052260 | 8.90 | 9.58 | Formate/nitrite transporter | Ballester et al., 2015 (72 h) |
| PEX1_058130 | 7.56 | 8.10 | MFS general substrate transporter |  |
| PEX1_029250 | 4.85 | 6.91 | High affinity zinc ion transporter | Ballester et al., 2015 (72 h) |
| PEX1_045460 | 7.00 | 6.56 | Related to OPT1-High-affinity glutathione transporter |  |
| PEX1_105680 | 5.29 | 6.43 | Related to GABA permease | Ballester et al., 2015 (48 h) |
| PEX1_061510 | 2.72 | 5.55 | Allantoin transport |  |
| PEX1_085740 | 4.23 | 5.29 | Ammonium transporter | Ballester et al., 2015 (48 h) |
| PEX1_036500 | 6.15 | 5.15 | High affinity nitrate transporter NrtB |  |
| PEX1_087660 | 5.07 | 5.11 | ABC Pleiotropic drug resistance protein | Barad et al., 2016 |
| PEX1_047280 | 3.64 | 5.10 | Amino acid transporter |  |
| PEX1_058750 | 5.21 | 4.43 | Multi antimicrobial extrusion protein MatE |  |
| PEX1_051580 | 2.42 | 4.38 | Nucleobase transmembrane transporter |  |
| PEX1_025540 | 5.09 | 4.37 | ABC Multidrug resistance protein | Ballester et al., 2015 (48 h) |
| PEX1_074780 | 4.28 | 3.73 | MFS general substrate transporter |  |
| **Virulence factors (GO unspecified)** | | | | |
| Proteases | | | | |
| PEX1_086060 | 8.24 | 7.93 | Peptidase S28, Endoprotease Endo-Pro-Aspergillus niger | Ballester et al., 2015 (48 h) |
| PEX1_073500 | 6.10 | 5.86 | Protease S8 tripeptidyl peptidase I (Secreted protein) | Ballester et al., 2015 (48 h); Levin et al., 2019 |
| PEX1_081750 | 6.33 | 5.36 | Aspergillopepsin | Ballester et al., 2015 (48 h) |
| PEX1_031490 | 4.58 | 2.46 | Aspartic proteinase aspergillopepsin Pep4 | López‐Pérez et al., 2015 |
| **CWDE - Pectinases** | | | | |
| PEX1_010660 | 11.68 | 13.33 | Pectin methylesterase | Wang et al., 2019 |
| PEX1_085620 | 11.36 | 10.28 | Pectin lyase fold/virulence factor |  |
| PEX1_103630 | 7.07 | 8.37 | Cellulose-binding beta-glucosidase | Wang et al., 2019 |
| PEX1_083670 | 7.17 | 6.81 | Pectin lyase fold/virulence factor | Ballester et al., 2015; Zhou et al., 2022 |
| PEX1_055640 | 6.43 | 4.81 | Endopolygalacturonase PGb | Ballester et al., 2015 |
| PEX1_014680 | 5.00 | 3.34 | Extracellular exo-polygalacturonase | Ballester et al., 2015; Sánchez et al., 2003 |
| **CWDE – Glycoside hydrolase** | | | | |
| PEX1_052930 | 6.76 | 7.11 | Alpha-glucanase |  |
| PEX1_081540 | 6.59 | 5.96 | Xyloglucan-specific endo-beta-1 | Ballester et al., 2015; Wang et al., 2019 |
| PEX1_107200 | 6.89 | 5.72 | Extracellular endoglucanase/cellulase | Ballester et al., 2015; Wang et al., 2019 |
| PEX1_063220 | 7.04 | 4.85 | Extracellular invertase (sucrose hydrolyzing enzyme) | Ballester et al., 2015 (48 h) |
| PEX1_041340 | 6.31 | 4.73 | Concanavalin A-like lectin/glucanase, subgroup |  |
| PEX1_055260 | 5.26 | 4.52 | Glycoside hydrolase, chitinase active site | Wang et al., 2019 |
| PEX1_083500 | 4.57 | 4.40 | Glycoside hydrolase-type carbohydrate-binding, subgroup |  |
| PEX1_085560 | 5.69 | 4.20 | Endo-1,4-beta-xylanase B | Ballester et al., 2015 |
| **Trascription factors** | | | | |
| PEX1_025180 | 4.35 | 6.02 | Arginine metabolism regulation protein Arg81 |  |
| PEX1_060030 | 5.84 | 4.91 | Zinc finger Msn4 |  |
| **Hypothetical proteins with N terminal signal peptide (predicted effectors)** | | | | |
| PEX1_077000 | 15.73 | 14.80 | ArfGap-domain-containing protein |  |
| PEX1_085630 | 12.45 | 11.59 | CFEM domain-containing protein |  |
| PEX1_096670 | 12.32 | 11.19 | Hypothetical protein | Ballester et al., 2015; Sánchez et al., 2003 |
| PEX1_077010 | 11.66 | 10.81 | Hypothetical protein | Ballester et al., 2015 |
